# Hierarchy of anomalies in the simple rose model of water

**DOI:** 10.1016/j.molliq.2023.122274

**Published:** 2023-06-08

**Authors:** Peter Ogrin, Tomaz Urbic

**Affiliations:** Faculty of Chemistry and Chemical Technology, University of Ljubljana, Vecna Pot 113, SI-1000 Ljubljana, Slovenia

## Abstract

The density, diffusion, and structural anomalies of the simple two-dimensional model of water were determined by Monte Carlo simulations. The rose model was used which is a very simple model for explaining the origin of water properties. Rose water molecules are modelled as two-dimensional Lennard-Jones disks with rose potentials for orientation dependent pairwise interactions mimicking formations of hydrogen bonds. The model can be seen also as a variance of silica-like models. Two parameters of potential in this work were selected in a way that (1) the model exhibits similar properties to Mercedes-Benz (MB) water model; and (2) that the model has real-like properties of water. Beside the known thermodynamic anomaly for the model we also found diffusion and structural anomalies. The orientational order parameters were calculated and maximum encountered for three and six-fold symmetry. For the MB parametrization, the anomalies occur in hierarchy order, which is a slight variation of the hierarchy order in real water. The diffusion anomaly region is the innermost in the hierarchy while for water it is the density anomaly region. In case of real water parametrization the most inner is the structural anomaly.

## 1. Introduction

The importance of water in different fields is widely accepted, however, sometimes, due to water’s abundance, the properties of water are treated as something normal for liquid. Yet, water exhibits many anomalous properties, which are of great importance, as they are crucial for many applications in which water is used. The most widely known anomalous property of water is density maximum at 4 °*C* at 1 atm. Due to this anomaly cold liquid water is denser than ice, thus ice floats on water and water freezes from top to bottom. This enables survival of organisms in water at temperatures below water’s freezing point. There are many more anomalous properties, such as high heat capacity, negative thermal expansion coefficient, high surface tension, etc., that are crucial for water’s role in life on Earth or it’s use in industry. Most of water’s anomalous properties appear at low temperatures where orientationaly ordered hydrogen bonds are formed between water molecules, which are the reason for these anomalous properties. At higher temperatures when the number of hydrogen bonds is substantially reduced water exhibits properties of normal liquid.

However, water is not the only liquid that exhibits anomalous properties. Anomalous properties similar to water’s were also found in liquid *GeO*_2_ [1], liquid silica [2,3], liquid silicon [4], liquid *BeF*_2_ [5,6]. The common thing to mention anomalous liquids is that they have tetrahedral structure [7]. In case of water oxygen atoms are tetrahedrally coordinated with hydrogen atoms as bridges between them, in *SiO*_2_ silicon atoms are tetrahedrally coordinated and oxygen atoms form bridges between them, and in silicon there are no bridge atoms, however silicon atoms still from tetrahedral coordination. Anomalous properties were also found in liquids that don’t form tetrahedral configurations or any other orientationally highly ordered bonding. Such liquids are for example sulphur [8], tellurium [9], gallium [10], germanium [11].

Anomalous properties, that expand over larger region of conditions and can be hierarchically ordered by covered area in density-temperature or pressure-temperature diagram, are density (contraction of liquid upon heating), diffusion (larger diffusion upon increased pressure) [12,13] and structural anomaly (order decreases when pressure increases) [14]. In water hierarchical order of anomalies is following: the most outer region is occupied by structural anomaly, which encloses a region of diffusion anomaly, which encloses a region of density anomaly [14]. However another common hierarchy that appears in *SiO*_2_ and some other liquids (for example *BeF*_2_ [15]) is when the most outer is region of diffusion anomaly, following by structural anomaly and most inner is again density anomaly [16].

In order to model water’s properties in different systems, many water models were developed which differ in their complexity and accuracy. The most widely used water models, that are usually used in computer simulations when water is present as a solvent in the system, are SPC [17] and TIP [18,19] models. In these models water molecules are atomistically realistic, models consist both from oxygen and hydrogen atoms with different parametrisations and differently placed charges. Even thou the aim of all water models is to model properties of water, not all water models have water-like hierarchy of anomalies, in fact many of water models exhibit silica-like hierarchy of anomalies. For example rigid-body models such as SPC/E [14], and TIP4P/2005 [20] exhibit water-like hierarchy of anomalies, while mW water model exhibits silica-like hierarchy [11]. The reason for difference between water-like and silica like hierarchy is not well understood. There were ideas that water-like hierarchy, where structural anomaly dominates over other anomalies, may appear when there is a strong correlation between translational order and tetrahedral order [11]. However, in case of silicon modelled with Stillinger-Weber potential there is silica-like hierarchy, even thou, the correlation between translational and tetrahedral order is high [21].

Even some isotropic models exhibit water like anomalies [22,23]. The Stell-Hammer potential has the same hierarchy of anomalies as water. In case of core-softened model the parametrisation of the model can change the hierarchy of anomalies from water-like to silica-like [24–26]. The transition from water-like to silica-like hierarchy occurs when depth of attractive part of core-softened potential is increased. Moreover, when increasing width of the repulsive shoulder in the potential diffusion and density anomaly order can be inverted. Even thou, some of these isotropic models exhibit water-like anomalies, the reason for them is different as in water. These models exhibit water-like anomalous properties due to two length scales – the interaction potential has two characteristic distances at which interacting molecules are likely to be located.

The middlepoint between atomistically realistic water models and core-softened water-like models are some simple water models, such as Mercedes-Benz (MB) [27,28] water model and rose water model [29]. Both of these models are simple two-dimensional models where water molecules are modelled as Lennard-Jones disks with added orientation-dependent hydrogen-bonding potential. The advantages of such simplified water models are greater insight into properties of the model, while being computationally less demanding. Due to computational effectiveness broader range of conditions can be explored in shorter time. Moreover, such simple models can be used in studying and developing analytical theories and methods such as integral equation theory [30–32] and thermodynamic perturbation theory [33–35]. It has also been show that MB model exhibits many of water anomalous properties, such as density anomaly, negative thermal expansion coefficient, hydrophobic effect [28,36–39]. Moreover the hierarchy of anomalies of MB model was also determined [40], where as, the hierarchy of MB model is neather water-like nor silica-like.

Rose water model has been developed as mimic of MB model. In rose model combination of sinusoidal functions in polar coordinates also known as rose functions are used to model hydrogen bonding potential between molecules. Rose model is also computationally more efficient than MB model. Properties of rose model are similar to MB model, however rose model is quite flexible, as parameters can be changed to model molecules with different properties. Comparison between MB and rose model has been made using Monte Carlo (MC) simulations, thermodynamic perturbation theory (TPT) and integral equation theory (IET) [41]. Furthermore, behaviour of rose water particles in porous media was studied using IET an MC [42], liquid-vapour line and percolation line of rose model were also determined using TPT and MC [43]. Interest in simplified models is due to insights that are not obtainable from all-atom computer simulations. Simpler models are more flexible in providing insights and illuminating concepts, and they do not require big computer resources. Our interest in using the rose model is that it serves like MB model as a testbed for developing analytical theories that might ultimately be useful for more realistic models. Another advantage of the rose model, compared to the more realistic water models, is that the underlying physical principles can be more readily explored and visualized in two dimensions.

In this work we studied anomalies of rose water model and their hierarchy. Similar study has been already done for MB model [40], so one of our goals was also to identify possible differences in anomalous properties of rose and MB models. In MB model hierarchy of anomalies is following: in the most inner region is diffusion anomaly, enclosed by density anomaly, and the most outer is structural anomaly. This is different as in water or in silica.

The paper is organized in the following way. In the next section we introduce the model used in this work (Sec. 2). In Sec. 3 information about Monte Carlo simulations are written. The results with discussion are provided in Sec. 4 and summarized in Sec. 5.

## 2. The model

The Rose water model is a simple two-dimensional water model, in which rose functions are used to describe hydrogen bonds [29]. Each water molecule is represented by a two-dimensional Lennard-Jones disk with hydrogen bonding potential added in order to be able to form hydrogen bonds:

(1) $$U{\left({\vec{X}}_{i},{\vec{X}}_{j}\right)}_{\text{rose }}=U_{LJ}\left(r_{ij}\right)+U_{HB}\left({\vec{X}}_{i},{\vec{X}}_{j}\right),$$

where *r*_*ij*_ is distance between centres of molecules *i* and *j*, ${\vec{X}}_{i}$ and ${\vec{X}}_{j}$ are vectors of positions and orientations of molecules *i* and *j*. LJ part of the potential has a standard form:

(2) $$U_{LJ}\left(r_{ij}\right)=4\epsilon_{LJ}\left({\left(\frac{\sigma_{LJ}}{r_{ij}}\right)}^{12}-{\left(\frac{\sigma_{LJ}}{r_{ij}}\right)}^{6}\right).$$

The hydrogen bonding pair potential is a sum of two separate contributions, while the contribution of each molecule to hydrogen bonding potential is independent of the contribution from other molecule in pair interaction

(3) $$U_{HB}\left({\vec{X}}_{i},{\vec{X}}_{j}\right)=U_{HB}\left(\vec{r_{ij}}\right)+U_{HB}\left(\vec{r_{ji}}\right).$$

Therefore, there is a possibility of a “half” hydrogen bond, which means that one molecule forms a hydrogen bond with the second molecule, while at the same time the second molecule doesn’t form hydrogen bond with the first one. The probability of the formation of such a “half” bond is, however, lower than probability of the formation of the full hydrogen bond. Each molecule’s independent contribution consists of a product of two terms, the first term (*s*(*r*_*ij*_)) is dependent on the distance between the centres of molecules. The second term (*U*(*θ*_*ij*_)) is dependent on the orientation of the molecule.


(4) $$U_{HB}\left(\vec{r_{ij}}\right)=\frac{\epsilon_{HB}}{2}*s\left(r_{ij}\right)*U\left(\theta_{ij}\right),$$

$\vec{r_{ij}}$ is a vector between molecules *i* and *j* oriented in the body frame of molecule *i*, *r*_*ij*_ is the length of this vector, and *θ*_*ij*_ angle of orientation of the vector in body frame of molecule *i*. *Є*_*HB*_ is HB energy parameter. In order to normalize HB energy to maximal value of 1, $\frac{1}{2}$ must be placed into equation (4). The value of orientational term of potential energy of molecule *i* depends on where molecule *j* is placed in respect to body frame of molecule *i*, this means that it depends on how molecule *i* is oriented in respect of molecule *j*. To describe orientational dependency 3-petal rose function in polar coordinate system are used:

(5) $$U\left(\theta_{ij}\right)=a_{2}{\text{sin}}^{2}\left(3\theta_{ij}\right)+a_{1}\text{sin}(3\theta_{ij}),$$

where *a*_1_ and *a*_2_ are coefficients determining amplitude of contributions of two sinusoidal terms and consequently determine the shape of angular-dependent part of the potential. The function can be also written in cartesian coordinates as:

(6) $$U_{HB}\left(\vec{r_{ij}}\right)=\frac{\epsilon_{HB}}{2}*s\left(r_{ij}\right)*\left(a_{2}*\frac{{\left(3x_{ij}^{2}y_{ij}-y_{ij}^{3}\right)}^{2}}{r_{ij}^{6}}+a_{1}*\frac{3x_{ij}^{2}y_{ij}-y_{ij}^{3}}{r_{ij}^{3}}\right)$$

where $r_{ij}=\sqrt{x_{ij}^{2}+y_{ij}^{2}}$, *x*_*ij*_ and *y*_*ij*_ are cartesian coordinates of molecule *j* in body frame of molecule *i*.

For distance dependent term double-sided cubic switching function is used:

$$s\left(r_{ij}\right)=\{\begin{array}{ll} 0, & r_{ij}<r_{l}\\ \frac{\left(r_{l}+2r_{ij}-3r_{HB}\right){\left(r_{l}-r_{ij}\right)}^{2}}{{\left(r_{l}-r_{HB}\right)}^{3}}, & r_{l}\leq r_{ij}<r_{HB}\\ \frac{\left(r_{u}+2r_{ij}-3r_{HB}\right){\left(r_{u}-r_{ij}\right)}^{2}}{{\left(r_{u}-r_{HB}\right)}^{3}}, & r_{HB}\leq r_{ij}<r_{u}\\ 0, & r_{u}\leq r_{ij}, \end{array}$$

where *r*_*HB*_ is HB distance, *r*_*l*_ and *r*_*u*_ are lower and upper bound of HB. Switching function is symmetrical, since |*r*_*HB*_ - *r*_*l*_| = |*r*_*HB*_ - *r*_*u*_| = *r*_*FWHM*_, as *r*_*FWHM*_ is “full width at half maximum” of peak formed by function.

The parameters of potential in this work were selected in a way that the model exhibits similar properties to Mercedes-Benz water model [27,28], which exhibits many of water’s anomalous properties. We named this parametrisation MB parametrisation and it’s parameters are following: *Є*_*LJ*_ = 0.1, *σ*_*LJ*_ = 0.7, *Є*_*HB*_ = 1, *r*_*HB*_ = 1, *r*_*FWHM*_ = 0.35, *a*_1_ = 0.7, and *a*_2_ = −0.3. For this parametrisation the parameters of Lennard-Jones part of the potential are the same as in MB model. The parameters of hydrogen-bonding potential were selected in following way: we calculated density as function of temperature at pressure 0.19 for many different sets of parameters, and then visually decided which set of parameters produces temperature dependence of density that is the most similar to dependence of density in MB model, while also having prominent density maximum.

Some of volumetric properties (for example contraction of volume upon melting) of MB model are exaggerated in comparison to real experimental water due to exaggerated difference between *σ*_*LJ*_ and *r*_*HB*_. That is why we also used a second parametrisation which should exhibit more real-like properties of water than MB model where the difference is not exaggerated. Also correlation function is similar as experimental one without LJ prepeak. We named this parametrisation real parametrisation, the difference between real and MB parametrisations are in Lennard-Jones parameters of the model as in real parametrisation *Є*_*LJ*_ =0.2 and *σ*_*LJ*_ =0.890899. Due to these changes the position of LJ minimum and distance of hydrogen bond is the same, moreover, the contribution of LJ part of the potential towards interactions is larger than in MB parametrisation. Note that the parameters of both versions are different that in the initial rose model version [29].

## 3. Monte Carlo simulations

Monte Carlo computer simulations in canonical (NVT) ensemble with Metropolis algorithm were performed in order to determine anomalies of the rose water model and their hierarchy. We used periodic boundary conditions and minimum image convention to dismiss the effect of limited size of the system. In each step of the simulation one molecule was randomly selected to be translated and another molecule was randomly selected to be rotated. On average each molecule was translated and rotated once in a cycle. Simulations always started from random configurations, following by equilibration which was 1000000 cycles long. After that sampling part of simulations was performed, which consisted of 20 series each in length of 1000000 cycles. All simulated systems consisted of 200 rose particles. We have tested also bigger and smaller systems and no size effects were observed. During sampling phase different structural and thermodynamic quantities were calculated. Using virial equation pressure was calculated. Pseudo-diffusion coefficient was calculated from the mean square displacement. In these MC simulation runs, the dynamic adjustment of maximum displacement was turned off and was kept fixed for all instances.


(7) $$D^{*}=\underset{n\to \infty }{\text{lim}}\frac{\langle \Delta r{\left(n\right)}^{2}\rangle }{n},$$

(8) $$\langle \Delta r{\left(n\right)}^{2}\rangle =\langle {\left[\vec{r}(n)-{\vec{r}}_{0}\right]}^{2}\rangle ,$$

${\vec{r}}_{0}$ is the initial position of the particle and *n* is the number of the cycle. We calculated the mean square displacement as an average over all particles. For normal fluids pseudo-diffusion coefficient decreases monotonically as the density increases at constant temperature.

Structural anomalies were also determined. First, translational order parameter *t* was calculated as:

(9) $$t=\rho^{1/2}\overset{r_{c}}{\underset{0}{\int }}\left|g(r)-1\right|dr$$

where *r* is distance between two particles, *g* is radial distribution function, *r*_*c*_ is the cut off distance which was set to half of simulation box length. The translational order parameter is a measurement of pair correlation in the system. Translational order parameter of normal fluids monotonically increases with density. Orientational order parameters were also calculated, we calculated the parameter with three-fold symmetry *q*_3_ and parameter with six-fold symmetry *q*_6_. The orientational order parameter *q*_*l*_ is calculated as an average of the absolute value of orientational order parameters of all particles in the system:

(10) $$q_{l}=\frac{1}{N}\overset{N}{\underset{i=1}{\sum }}\left|q_{l}^{i}\right|$$

where *N* is number of particles and $q_{l}^{i}$ is orientational order parameter of one particle calculated as:

(11) $$q_{l}^{i}=\frac{1}{n_{j}}\underset{k}{\sum }\text{exp}\left(il\theta_{jk}\right),$$

where *n*_*j*_ represents the number of neighbour particles in the first atom shell of particle *j*, *l* is the number which specifies the type of symmetry, which is 3 or 6 in our case, and *θ*_*jk*_ is the angle between the vector connecting particles *j* and *k* and horizontal axis. The first atom shell of particle *j* is defined as particles that are located at distance lower than 1.2, which is the position of the first minimum after hydrogen-bonding peak in radial distribution function between rose particles.

Pair entropy was also calculated. Pair entropy is the dominant contribution to excess entropy [5,6,44–49]. Excess entropy can be expressed by the multiparticle correlation expansion as:

(12) $$s_{e}=s_{2}+s_{3}+\ldots +s_{n}$$

where *s*_*n*_ is n-particle correlation contribution to excess entropy. Pair entropy, *s*_2_, was calculated as:

(13) $$s_{2}=-\pi \rho \overset{L/2}{\underset{0}{\int }}[g(r)\text{ln}g(r)-g(r)+1]rdr,$$

where *L*∕2 is half of simulation box length, *ρ* is number density and *g*(*r*) is pair distribution function.

## 4. Results and discussion

All results are presented in reduced units, reduced in relation to the strength and length of an HB interaction. In this reduction, the HB energy parameter *Є*_*HB*_ was used to normalize temperature and excess internal enthalpy ($A^{*}=\frac{A}{\left|\varepsilon_{HB}\right|}$, $T^{*}=\frac{k_{B}*T}{\left|\varepsilon_{HB}\right|}$), while all distances were normalized with characteristic length of the hydrogen bond $r_{HB}\left(r^{*}=\frac{r}{r_{HB}}\right)$.

**Fig. 1.**
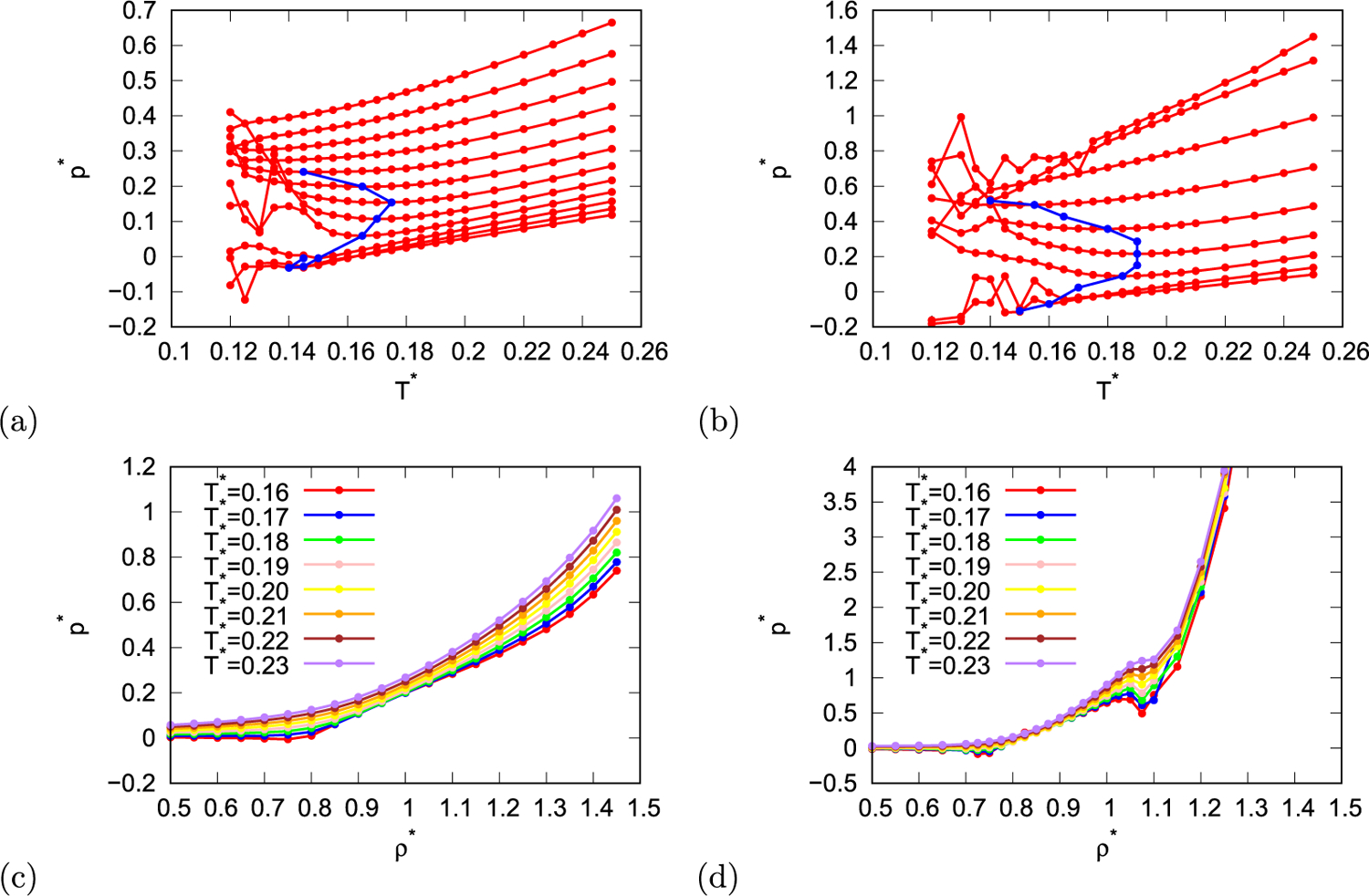
First row: red lines show temperature dependence of pressure of rose model with (a) MB parametrisation and (b) real parametrisation at different constant densities (0.7, 0.75, 0.8, 0.85, 0.9, 0.95, 1.0, 1.05, 1.1, 1.15, 1.2, 1.25 (last three only for (a)), blue line indicates positions of density maximums at different temperatures and pressures. Second row: pressure as function of density for different temperatures for rose model with (c) MB parametrisation and (d) real parametrisation.

First, the density anomaly of rose water model was explored. At some constant pressures density as function of temperature has maximum (like real water at 4 °*C* and 1 atm). The temperature at which density maximum is present changes as function of pressure. The temperature of maximum density at constant pressure is the same as temperature of pressure minimum at constant density, therefore, position of density maximum can be also located by locating pressure minimum at constant densities. In Fig. 1 (a) and (b) pressure as function of temperature is shown with red lines for multiple different densities and both parametrisations of the model. At some densities local minima are present in pressure as function of temperature, as mentioned before these minima are at the same temperatures as density maxima, the positions of these extrema are marked with blue points and line in Fig. 1 (a) and (b). In other words, blue line indicates density anomaly. The density anomaly of rose model with MB parametrisation is located at temperatures below 0.175, and at density between 0.7 and 1.1, which corresponds to pressures between 0 and 0.3. For real parametrisation of the model the density anomaly is located on larger area of conditions, which is up to temperature 0.19 and between densities 0.725 and 1.05, which corresponds to virial pressures between −0.11 and 0.52.

The reason for density anomaly is in structure of rose water model. At low temperature at low pressures when rose water is in solid state, particles of rose water model are connected with hydrogen bonds and form highly ordered hexagonal lattice with vacancies between rose particles. As temperature increases and water becomes liquid structure becomes more flexible, however, most of hydrogen bonds are still present in the system. The structure is less ordered in liquid state and besides hexagons rose particles also form many pentagons and heptagons. Due to flexibility of network of hydrogen bonds some vacancies that were empty in solid state are now occupied by particles, thus the density of the system is increased. Upon further increase in temperature the number of hydrogen bonds in the systems decreases, thus network of hydrogen bonds starts to break down and the system becomes more distorted, at this point density of the system is decreasing. As the number of hydrogen bonds in the system lowers the rose particles start to behave more like normal liquids.

In Fig. 1 (c) and (d) pressure as function of density is shown for different temperatures. The pressure generally increases with increasing pressure, however, the slope of pressure as function of density is different at regions where the model exhibits anomalous properties (as will be shown in the rest of the paper).

**Fig. 2.**
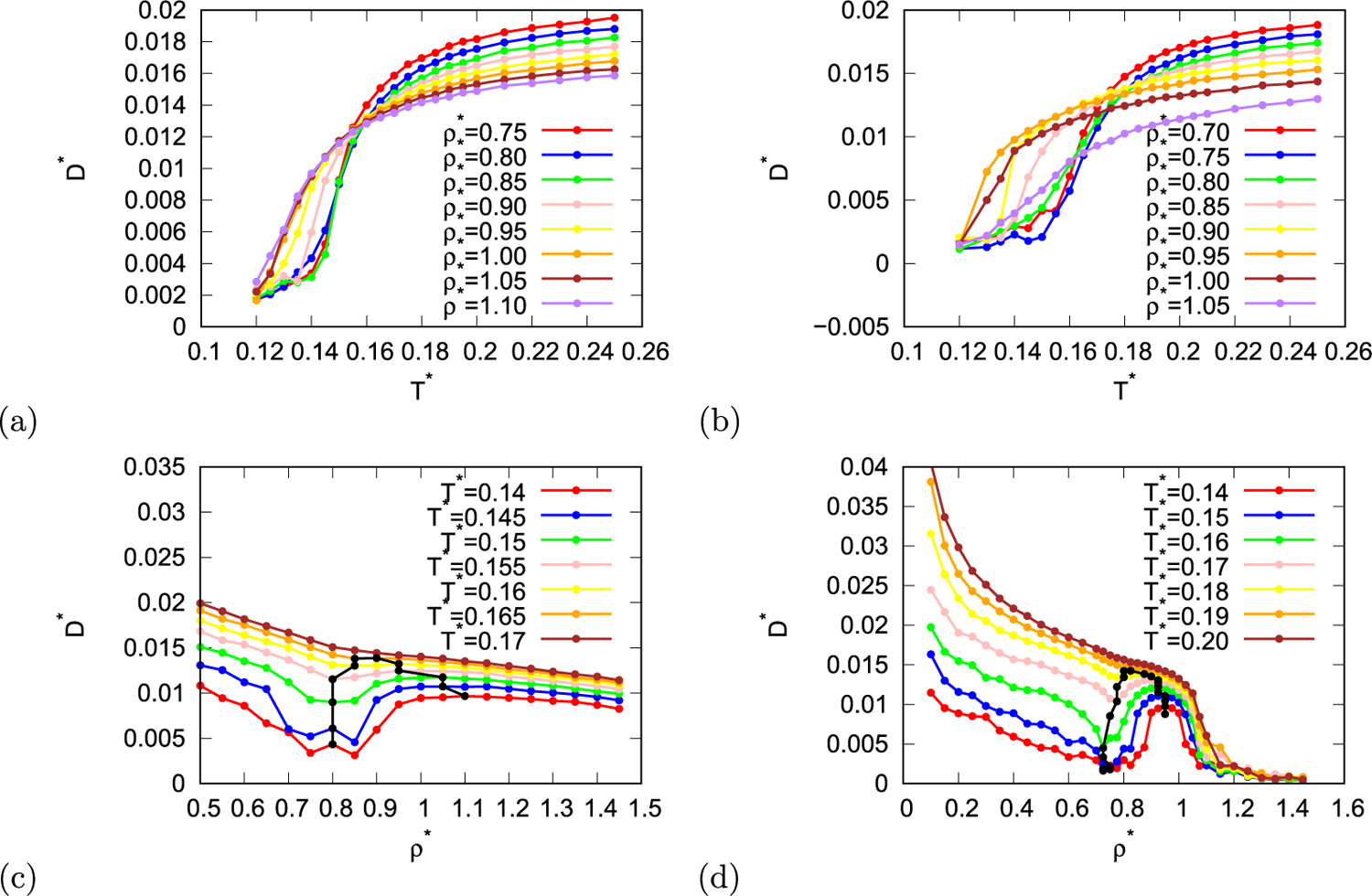
Pseudo-diffusion coefficient as function of temperature (first row) and density (second row) for MB parametrisation (first column) and real parametrisation (second column) of the rose model. Black line indicates local maxima and minima of pseudo-diffusion coefficient.

**Fig. 3.**
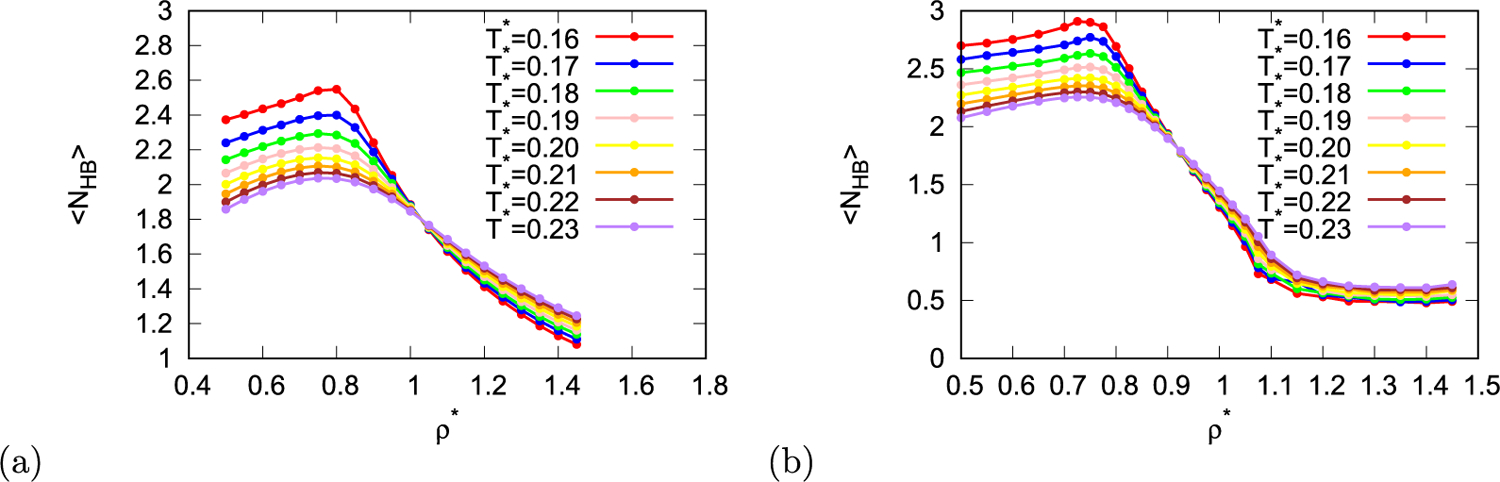
Average number of hydrogen bonds per molecule as function of density for different temperatures for rose model with (a) MB parametrisation and (b) real parametrisation.

Next, pseudo-diffusion coefficient was calculated from displacement of particles during MC simulation when maximum particle steep was constant. To calculate real diffusion coefficient molecular dynamics simulations are required as diffusion coefficient is time dependent quantity, however, even thou MC simulations don’t have time dimension, MC simulations can be used to obtain pseudo-diffusion coefficient which is proportional to real diffusion coefficient. This is done in such way that at fixed maximum step the averaged displacement is plotted as function of average number of attempted moves per particle. As we are interested in relative changes of diffusion coefficient at constant temperatures, pseudo-diffusion coefficient is sufficient for our needs. In Fig. 2 (a) and (b) pseudo-diffusion coefficient is shown as function of temperature at different densities for both parametrisations of the rose model. The diffusion coefficient increases with increased temperatures. At higher density this increase is slower due to steric constrains. In normal liquids diffusion coefficient decreases with increasing density at constant temperature. The reason for this is that at higher density there is less free space in the system, thus the movement of particles is more obstructed, which decreases length of path that particle can travel in certain time and thus decrease the diffusion coefficient. From Fig. 2 (c) and (d) it is seen that rose water model exhibits diffusion anomaly, which for MB parametrisation of the model lies at temperature lower than 0.165 and for real parametrisation of the model at temperatures below 0.19. In region where this anomaly is located, diffusion coefficient first decreases with increasing density, then after it reaches local minimum it increases. Upon further increase in density local maximum of diffusion coefficient is reached, and after that the coefficient decreases as in normal liquids. This anomaly region is also present in the density range between the high and low density ices. The reason for such behaviour is that at low density rose particles are connected with hydrogen bonds (Fig. 3), however, due to low density there are “holes” in the structure and not all particles form three hydrogen bonds, therefore the structure is not perfect hexagonal lattice. As the density increases, the average number of hydrogen bonds increases and the structure becomes close to perfect hexagonal lattice. At this point most particles are fixated in the lattice thus minimum in pseudo-diffusion coefficient is reached. Upon further increase of the density water molecules occupy empty space within the hexagons and diffusion starts to increase since these molecules are less bonded than molecules forming HB networks and can move more easily (Fig. 3). Upon further increase in density the system becomes more and more crowded, formation of hydrogen bonds becomes unfavourable, at his point the diffusion coefficient decreases due to obstructed mobility of the particles in a crowded system. What is unexpected is that this diffusion anomalous region lies within the density anomalous region like in MB model [40]. In water-like models and in silica-like models the density anomalous region is the most inner region. The reason for this might be the dimensionality of the system.

**Fig. 4.**
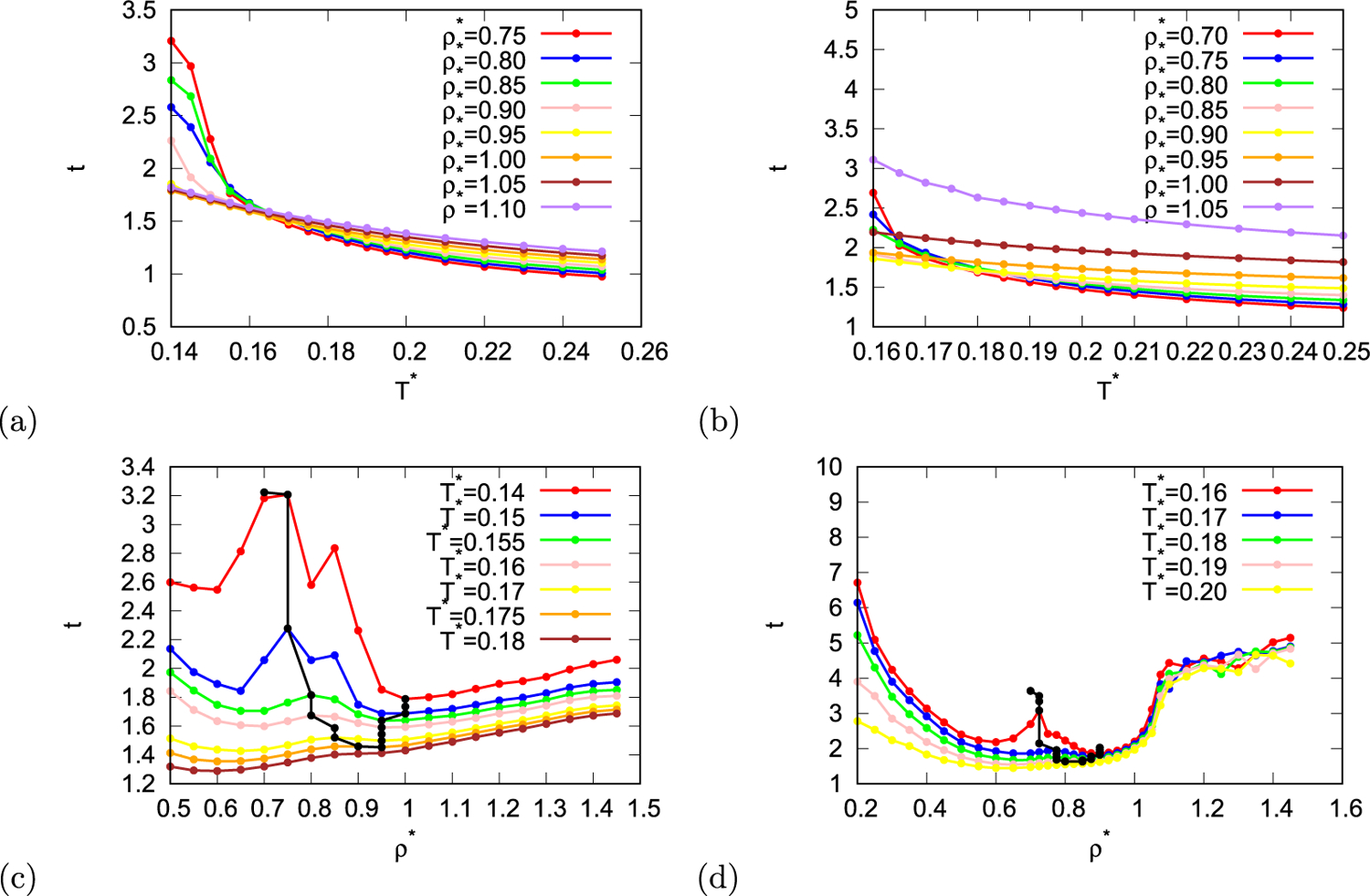
Translational order parameter as function of temperature (first row) and density (second row) for MB parametrisation (first column) and real parametrisation (second column) of the rose model. Black line indicates local maxima and minima of translational order parameter.

Structural ordering in the system was studied using three order parameters - translational, three- and six-fold orientational order parameters. Translational order parameter of normal liquids increases with increasing density, however in case of rose model there are local extrema in translational order parameter as function of density. In Fig. 4 (a) and (b) translational order parameter is shown as function of temperature and in Fig. 4 (c) and (d) as a function of density. As the temperature increases the effect of hydrogen bonds and other interactions on structure decreases, while movement of molecules increases due to thermal energy of molecules. This results in decrease of translational order parameter with temperature. Lower temperatures have bigger drop which shows as crossover of the lines. On the other hand translational order parameter as function of density is not so monotonic. With black line local minima and maxima of translational order parameter as function of density are indicated in Fig. 4 (c) and (d). The region within the limits of black line is anomalous structure region and it appears at temperatures below 0.175 for MB parametrisation of the model and below temperature 0.19 for real parametrisation of the model.

**Fig. 5.**
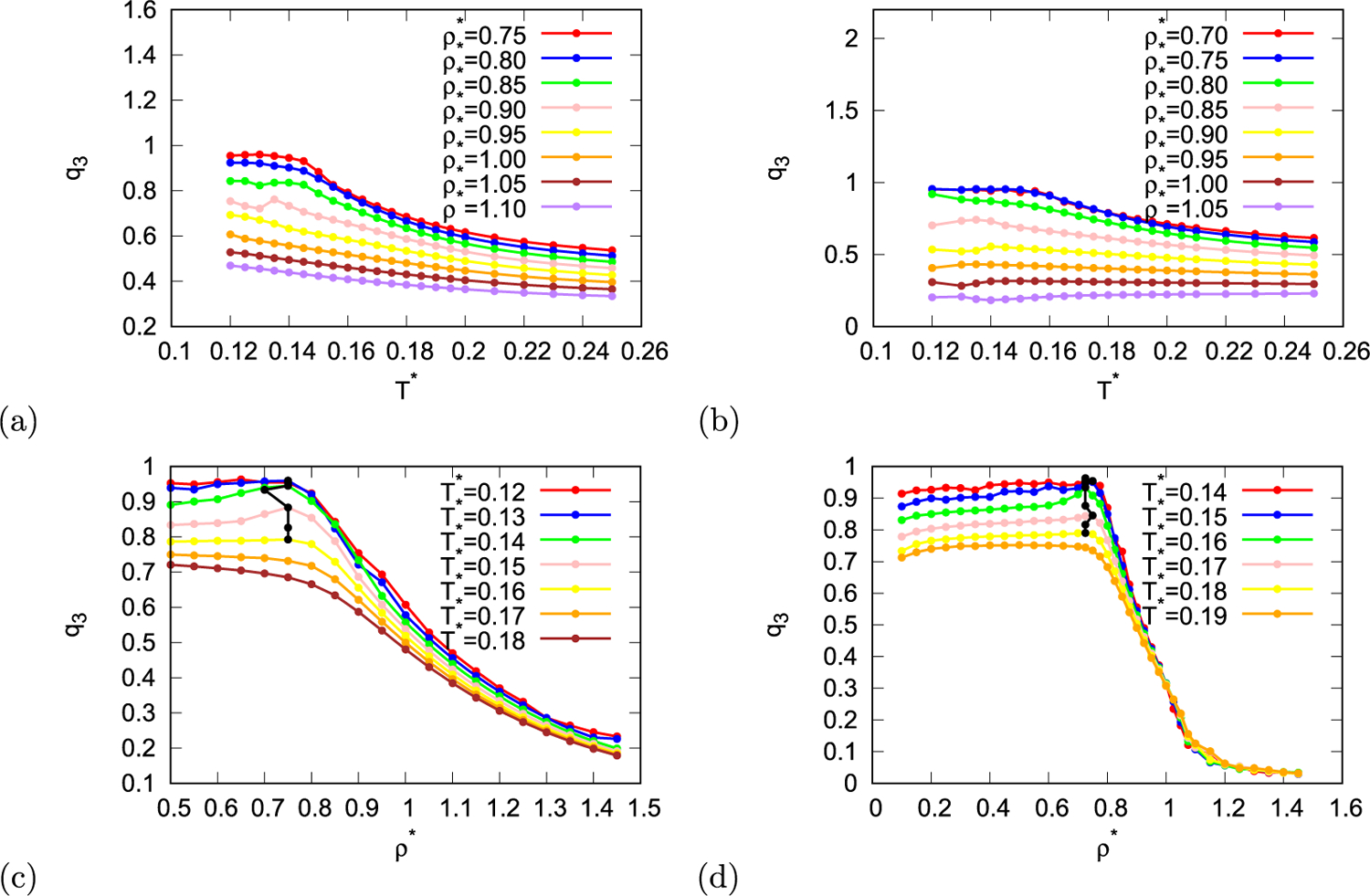
Orientational order parameter with three-fold symmetry as function of temperature (first row) and density (second row) for MB parametrisation (first column) and real parametrisation (second column) of the rose model. Black line indicates local maxima and minima of orientational order parameter with three-fold symmetry.

**Fig. 6.**
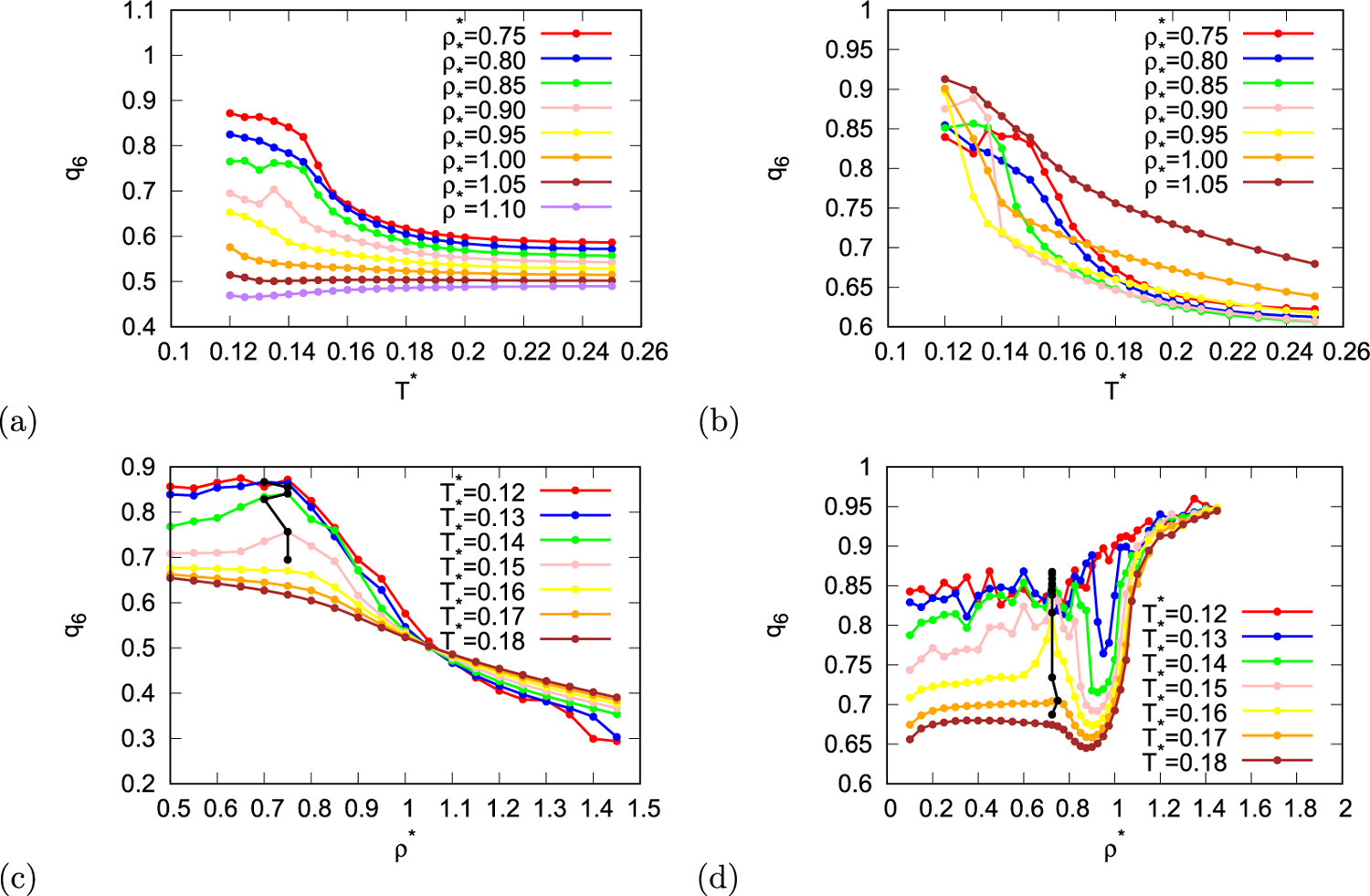
Orientational order parameter with six-fold symmetry as function of temperature (first row) and density (second row) for MB parametrisation (first column) and real parametrisation (second column) of the rose model. Black line indicates local maxima and minima of orientational order parameter with six-fold symmetry.

Both orientational order parameters also exhibit some anomalous behaviour. In Fig. 5 (a) and (b) three-fold orientational order parameter is plotted as function of temperature for different densities and in Fig. 5 (c) and (d) as function of density for different temperatures. In the same way six-fold orientational order parameter is plotted in Fig. 6. Three-fold orientational order parameters as function of density have maximum at density around 0.75 for both parametrisations, however, this maximum appears only at temperatures lower than 0.16 for MB parametrisation and at temperatures lower than 0.18 for real parametrisation. In case of MB parametrisation of the rose model six-fold orientational parameter has similar density dependency than three-fold parameter, and it also exhibits maximum at density close to 0.75 for temperatures below 0.16. However, in case of rose model with real parametrisation density dependence of six-fold parameter appears completely different. When density is increasing from low values six-fold parameter is first relatively constant, then at some temperatures it increases into maximum and soon after that decreases into minimum. As the density is increased even more the six-fold parameter increases and reaches plateau. We can still determine the position of the first maximum which appears around density 0.725 at temperatures below 0.175.

**Fig. 7.**
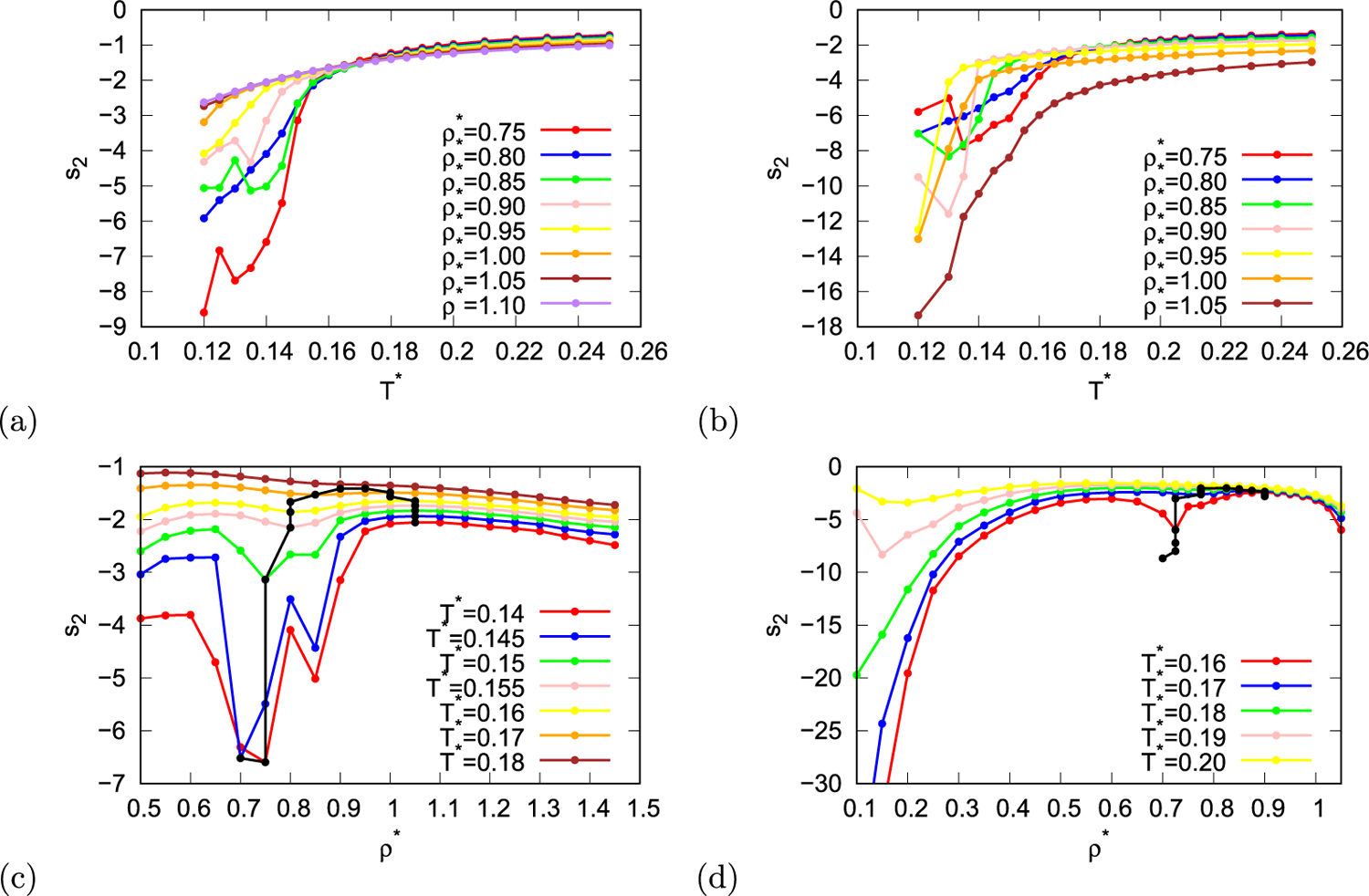
The pair entropy as function of temperature (first row) and density (second row) for MB parametrisation (first column) and real parametrisation (second column) of the rose model. Black line indicates local maxima and minima of the pair entropy.

Next, entropy anomaly was studied. For normal liquids entropy decreases if the liquid is compressed, therefore, if in certain region entropy of liquid increases upon compression, entropy anomaly is present. The ideal contribution to entropy monotonically decreases with increasing density, consequently, in anomalous region excess entropy should increase with increasing density. We calculated pair entropy which is, as mentioned before, the major contribution to excess entropy. The temperature dependence of pair entropy is shown in Fig. 7 (a) and (b) and density dependency is shown in Fig. 7 (c) and (d). With black line positions of local maxima and minima of pair entropy as function of density are indicated in Fig. 7 (c) and (d). The region beneath black line exhibits entropy anomaly as the pair entropy inside the region increases with increasing density. In case of MB parametrisation of the model this region lies at temperatures below 0.175 and in case of real parametrisation at temperatures below 0.185.

**Fig. 8.**
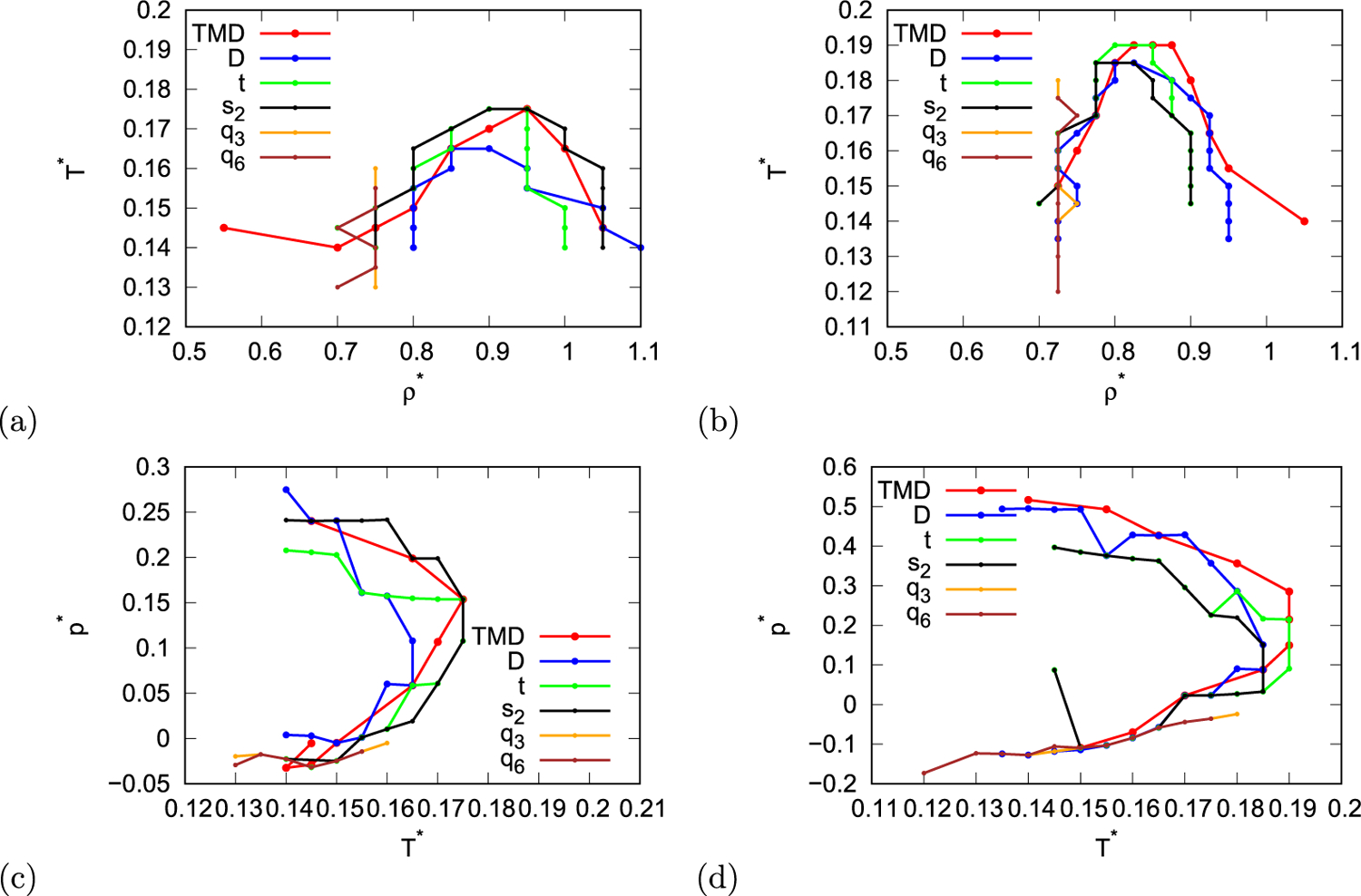
Position of all studied anomalies in temperature-density (first row) and pressure-temperature (second row) plane for MB parametrisation (first column) and real parametrisation (second column) of the model.

Fig. 8 shows anomalous regions of different properties for both parametrisations of the model. In case of MB parametrisations all anomalies appear at temperatures lower than 0.18, between density 0.7 and 1.1, and at pressure lower than 0.3. However, in case of real parametrisation anomalous regions extend to even higher temperatures. When MB parametrisation is used the most outer line is line that represents entropy anomaly, as entropy anomaly covers the largest part of region. A little smaller area of conditions exhibits structural anomaly (translational order), and in the region of similar size density anomaly is present, however, position of density anomaly is shifted more towards higher density/pressure. Diffusion anomaly is the most inner region. So in case of MB parametrisation the hierarchy of anomalies is neither water-like nor silica-like, as the order of anomalies is different from bot of them. However, the hierarchy of rose model is more similar to waterlike hierarchy as the diffusion anomaly is inside of structural anomaly region. On the other hand in case of real parametrisation the hierarchy of anomalies is different. The most outer region is region of density anomaly, then inside of it is diffusion anomaly region. The next line is line indicating structural anomaly (translational order), however, this region extends to temperatures higher than diffusion anomaly. The most inner region is region of entropy anomaly. Similar as MB parametrisation of rose model, real parametrisation of rose model also exhibits hierarchy of anomalies that doesn’t match neither with water-like nor silica-like hierarchy. However, when real parametrisation is used the region of diffusion anomaly expands comparing to region of diffusion anomaly in MB parametrised model. Therefore, real parametrisation exhibits a little more silica-like hierarchy (region of diffusion anomaly larger than region of structural anomaly) than MB parametrisation.

Rose water model was designed to mimic properties of Mercedes-Benz water model, so it is expected that anomalous properties of rose model are similar to those of MB model. The analysis of hierarchy of anomalies in this work was carried out similarly as previously for MB model [40], one of the aims of this work was also to identify differences in anomalous behaviour of rose and MB model. There is a big difference in the design of hydrogen bonding potential of these two models. In MB model formation of hydrogen bond depends on orientation of both particles in interaction, which means that if one particle is in unfavourable orientation there is no hydrogen bond between them. However, in rose model there is a possibility of half hydrogen bonds, due to independent contributions of orientation of interaction particles to hydrogen bonding potential. On the other hand the parameters of rose model were selected in a way that the properties of rose model are similar to MB model. Therefore, due to this difference there is a possibility that there could be some differences in anomalous behaviour of the models and it is important to identify if there are any. Comparing to anomalies of MB model [40], rose model with MB parametrisation has similar hierarchy of anomalies: in both models the most outer region is entropy anomaly, following by translational anomaly, then density anomaly and finally diffusion anomaly. So order in which different anomalies appear in Fig. 8 is the same as in MB model. Moreover, when studying separate anomalous properties similar trends can be observed in both models. However, the exact values of properties are not the same. The regions in which anomalous properties appear differ between models, for example the temperature below which anomalies are observed is mostly lower in case of rose model. So to sum up the hierarchical order of anomalies is the same, while the sizes of anomalous regions differ between models. On the other hand when real parametrisation of rose model is used different hierarchy of anomalies is obtained than in case of MB model, this hierarchy is also different then in real (experimental) water and silica.

All in all, rose water model exhibits anomalous properties that were identified in MB model with the same or different hierarchy of anomalies depending on parametrisation. Therefore, rose model is valid replacement for MB model, while being consiprationally less demanding and easier to modify.

## 5. Conclusions

We used Monte Carlo computer simulations in canonical ensemble in order to determine hierarchy of anomalies of rose water model. Rose water model is a simple water model that was designed to mimic properties of similar Mercedes-Benz water model, while being computationally more efficient. Both models consist of Lennard-Jones disks with added hydrogen bonding potential. The difference between models lies in the hydrogen bonding potential. Besides different functions used in the potential, the rose model also allows formation of “half” hydrogen bond - that is situation in which one particles in pair interaction forms hydrogen bond and other particle does not. In this work our aim was to analyse the hierarchy of anomalies in rose model and to identify any possible difference to anomalies previously determined for MB model. We used two different parametrisations of the rose model, one similar to MB model and one more similar to real-like water. The rose model exhibits density anomaly, structural anomaly, entropy anomaly and diffusion anomaly. The hierarchy of anomalies in case of MB-like parametrisation is following: the largest region belongs to entropy anomaly, following by structural and density anomaly and the smallest region belongs to diffusion anomaly. This hierarchy is the same as in MB model, however, the exact values of limits of different anomalous regions differ between the models. In case of real-like parametrisation the hierarchy is a little different: the largest region belongs to density anomaly, following by diffusion and structural anomalies and the most inner region belongs to entropy anomaly. Both parametrisations of rose model exhibit hierarchy of anomalies that is neither water-like nor silica-like. However MB parametrisation tends to lean more towards water-like hierarchy as the diffusion anomaly region is smaller than structural anomaly region, while real parametrisation leans more towards silica-like hierarchy as the diffusion anomaly region is larger than structural anomaly region. To conclude, rose model can be used as computationally less demanding alternative to MB model, while exhibiting semi-quantitatively similar anomalies, moreover the hierarchy of the anomalies can be manipulated by changing parametrisation of the model.
